# Sorrowing Poverty

**Published:** 1882-05

**Authors:** 


### SORROWING POVERTY.

THAT infant children should be starved to death deliberately in the great city of New York, is an almost incredible statement, except to the few, whose large intercourse with the world, has led them to the observation, that there is no meanness so unfathomable but some human wretch may be found who shall dive down and perpetrate it.

On a February day, within six squares of the palatial residences of Fifth Avenue, three infants were found, so abject and idiotic in expression that all trace of humanity seemed lost. "They could not cry, and so brutish were they, that when lifted from their cradles they merely gazed about, as puppies or kittens; none of them had any flesh on their bones."

Nearly two thousand children whose parents cannot take care of them, are constantly on the hands of the city; the helpless children of crime, of poverty, of infidelity and prostitution. About two hundred of the above are to be nursed, and are distributed over the city to women who profess to have lost their own children; of these two hundred, were the three above referred to.

We do not hold up to public reprobation the woman who engaged to take care of these three children, at the stipulated payment of one dollar a week each, the price paid for boarding dogs in —— street, for most likely she was poor and ignorant and perhaps herself at not a great remove from starvation.—Half frozen and famishing, the very best of us can't say what we would not do. Theoretical incorruptibility is the easiest of all easy things, to those who roll in wealth.

**PESTILENCE AND PLAGUE** have destroyed millions of lives. In long ages past, they have half-depopulated cities, and decimated empires. Whence came they? From human dereliction, as all other curses and calamities come, and not from the Almighty's hand, because he promised Noah, "I will not again curse the ground any more for man's sake," and, as we paraphrase the assertion, "let him be ever so bad." Plague is from a Greek word meaning "to blow," as if it came on the wings of the wind. Pestilence is the Latin word which means the same thing. The "Great Plague" visited London in 1665, and the next year a terrible fire laid a very large portion of it in ashes, since when it has not re-appeared there. Erasmus wrote of England three hundred and fifty years ago, in the days of "King Hal:" "The floors of their dwellings are strewn with green rushes, which are allowed to increase, layer upon layer, for twenty years together, covering up bones, crumbs from the table, and other filth; and to this, and the general dirty and slovenly habits amongst the people, may be ascribed the frequent plagues in England." And this in Henry the Eighth's day! What a disgusting idea! Yet, in our own time, filthy cellars, dirty kitchens, and foul, dark closets, are the unsuspected sources of habitual sickness to whole households.

